# Identification of Human Junctional Adhesion Molecule 1 as a Functional Receptor for the Hom-1 Calicivirus on Human Cells

**DOI:** 10.1128/mBio.00031-17

**Published:** 2017-02-14

**Authors:** Stanislav V. Sosnovtsev, Carlos Sandoval-Jaime, Gabriel I. Parra, Christine M. Tin, Ronald W. Jones, Jo Soden, Donna Barnes, Jim Freeth, Alvin W. Smith, Kim Y. Green

**Affiliations:** a Caliciviruses Section, Laboratory of Infectious Diseases, NIAID, NIH, Bethesda, Maryland, USA; b Laboratory for Calicivirus Studies, Oregon State University, Corvallis, Oregon, USA; c Retrogenix Ltd., Whaley Bridge, High Peak, United Kingdom; Baylor College of Medicine

## Abstract

The Hom-1 vesivirus was reported in 1998 following the inadvertent transmission of the animal calicivirus San Miguel sea lion virus to a human host in a laboratory. We characterized the Hom-1 strain and investigated the mechanism by which human cells could be infected. An expression library of 3,559 human plasma membrane proteins was screened for reactivity with Hom-1 virus-like particles, and a single interacting protein, human junctional adhesion molecule 1 (hJAM1), was identified. Transient expression of hJAM1 conferred susceptibility to Hom-1 infection on nonpermissive Chinese hamster ovary (CHO) cells. Virus infection was markedly inhibited when CHO cells stably expressing hJAM were pretreated with anti-hJAM1 monoclonal antibodies. Cell lines of human origin were tested for growth of Hom-1, and efficient replication was observed in HepG2, HuH7, and SK-CO15 cells. The three cell lines (of hepatic or intestinal origin) were confirmed to express hJAM1 on their surface, and clustered regularly interspaced short palindromic repeats/Cas9-mediated knockout of the hJAM1 gene in each line abolished Hom-1 propagation. Taken together, our data indicate that entry of the Hom-1 vesivirus into these permissive human cell lines is mediated by the plasma membrane protein hJAM1 as a functional receptor.

## IMPORTANCE

Vesiviruses, such as San Miguel sea lion virus and feline calicivirus, are typically associated with infection in animal hosts. Following the accidental infection of a laboratory worker with San Miguel sea lion virus, a related virus was isolated in cell culture and named Hom-1. In this study, we found that Hom-1 could be propagated in a number of human cell lines, making it the first calicivirus to replicate efficiently in cultured human cells. Screening of a library of human cell surface membrane proteins showed that the virus could utilize human junctional adhesion molecule 1 as a receptor to enter cells and initiate replication. The Hom-1 virus presents a new system for the study of calicivirus biology and species specificity.

## INTRODUCTION

Vesiviruses are small (~35-nm), nonenveloped, single-stranded RNA viruses belonging to the family* Caliciviridae*, which is currently divided into five genera: *Vesivirus*, *Lagovirus*, *Nebovirus*, *Sapovirus*, and *Norovirus*. The vesivirus RNA genome is organized into three major open reading frames (ORFs) (1). The nonstructural proteins are encoded within a large polyprotein in ORF1 beginning at the 5′ end of the genome and are released by proteolytic cleavage during replication. The ORF2 sequence encoding a capsid precursor protein is located toward the 3′ end of the genome and overlaps ORF3, which encodes a basic minor structural protein, VP2. The capsid precursor and VP2 proteins are expressed from an abundant subgenomic RNA in infected cells (2, 3). Maturation of the vesivirus major capsid protein VP1 involves proteolytic cleavage of the capsid precursor protein between the capsid leader sequence (LC) and VP1 by the same viral proteinase that mediates processing of the ORF1 polyprotein (4). The assembly of infectious calicivirus particles requires 180 monomers of the VP1 (5). Calicivirus VP1 can be structurally subdivided into two domains, the N-terminal shell (S) domain involved in the assembly of the icosahedral scaffold of the virion and the C-terminal protruding (P) domain that forms arch-like structures on the virion surface (5). The sequence variability of the latter domain defines the antigenic diversity of caliciviruses and is thought to be responsible for the marked differences in tropism among caliciviruses. Accordingly, the P domain contains virus neutralization epitopes and amino acid residues involved in attachment of the virus to cells (6, 7).

Vesiviruses infect a broad range of animal hosts and are associated with various chronic and acute illnesses (8). They cluster into three phylogenetically distinct groups. One group consists of feline calicivirus (FCV) strains, and a second includes canine calicivirus (CaCV) and related strains. The third and largest group (known as the “marine” vesiviruses) includes viruses closely related to the vesicular exanthema of swine virus (VESV), first associated with a foot-and-mouth disease-like syndrome in pigs in the United States in the 1930s (9). The transmission of a marine vesivirus, San Miguel sea lion virus (SMSV), to pigs has been observed experimentally, but with variable results (10, 11), and the frequency of interspecies transmission in nature remains unclear.

The zoonotic potential of these viruses is not known. It has been reported that marine vesiviruses can infect several primate species. VESV-related viruses have been isolated from pygmy chimpanzees, douc and silver leaf langurs, spider monkeys, and lowland gorillas (12–14). The only recorded case of vesivirus isolation from a human patient resulted from the apparent accidental infection of a laboratory worker (15). The researcher, working with CsCl-purified SMSV-5 virions, developed an influenza-like fever and vesicular lesions on all four extremities. The virus was isolated from one of the vesicles, and sequencing of the polymerase region showed a close relationship to the SMSV-5 strain studied in the laboratory (15). The virus was recovered in Vero cells and designated SMSV-5 Homosapien-1 or Hom-1. Here, we report the genetic characterization of this virus and show the ability of Hom-1 to replicate in several human cell lines of hepatic and intestinal origin. Using virus-like particles (VLPs), high-throughput screening of an expression library of human plasma membrane proteins (hPMPs), and clustered regularly interspaced short palindromic repeats (CRISPR)/Cas9 mutagenesis, we show that the Hom-1 vesivirus can interact with hJAM1 to enter cells and establish a productive infection.

## RESULTS

### Cell culture growth and genetic characterization of Hom-1 virus.

**FIG 1:**
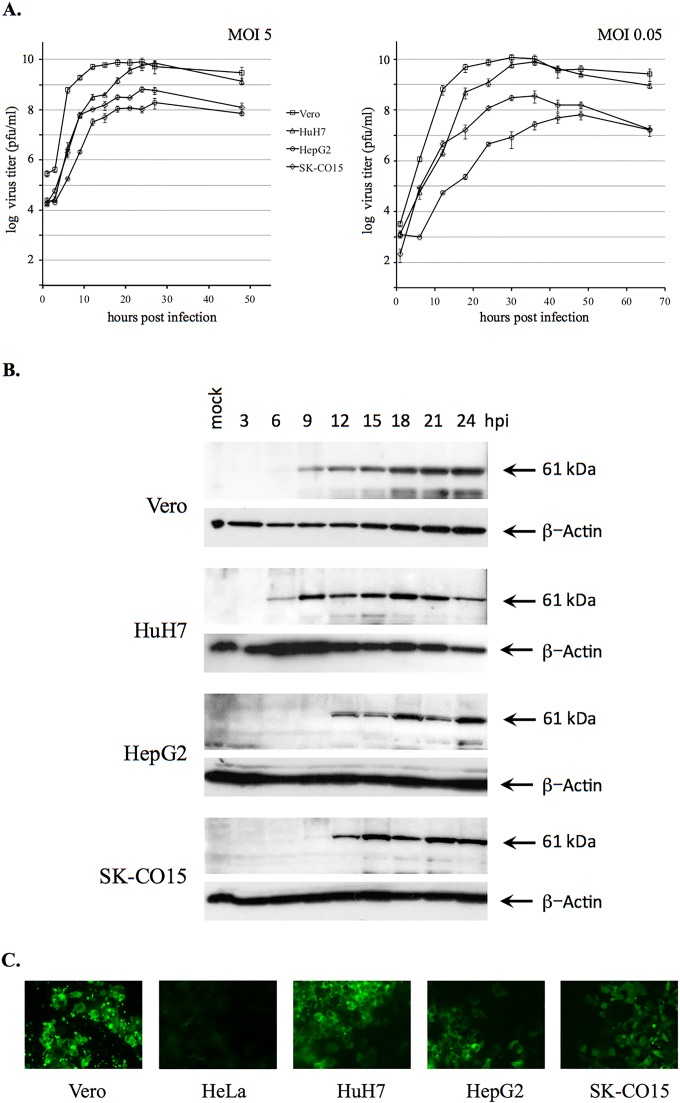
Different replication efficiencies of Hom-1 in Vero cells and different cell lines of human origin. (A) Growth kinetics of Hom-1 in Vero, HuH7, HepG2, and SK-CO15 cells were analyzed at MOIs of 5 and 0.05. Infected cells and growth medium were collected at various time points postinfection, and virus titers were determined by endpoint titration by plaque assay. The data represent the mean titer and standard error for each time point. (B) Western blot analysis of Hom-1 capsid protein synthesis in virus-infected cells. Cell monolayers in six-well plates (3 × 10^6^ to 4 × 10^6^ cells/well) were infected with Hom-1 at an MOI of 5, lysed with SDS sample buffer, and collected at different times postinfection. Proteins from the lysed samples were resolved by SDS-PAGE, transferred onto nitrocellulose membrane, and probed with anti-810 VLP serum. Probing with anti-β-actin antibodies served as a loading control. The 61.4-kDa capsid protein is indicated by an arrow. (C) Immunofluorescent detection of expression of the Hom-1 capsid protein in virus-infected cells. Cell monolayers were infected with ~100 PFU of Hom-1. At 48 hpi, infected cells were treated with 4% PFA and 0.1% Triton X-100 and incubated with guinea pig anti-810 VLP serum. Bound antibodies were detected with Alexa Fluor 488-conjugated secondary antibodies raised in goats against guinea pig IgG.

To initiate genetic characterization of the Hom-1 strain, the original cell culture stock of virus (15) was obtained from the ATCC under USDA permit 105439 and reamplified in Vero cells. In 16 to 24 h, Hom-1 infection induced a pronounced cytopathic effect (CPE) in the cells. The virus grew efficiently in Vero cells, reaching titers of >10^9^ PFU/ml at 16 to 20 h postinfection (hpi) (Fig. 1A). Hyperimmune serum raised against VLPs of Steller sea lion vesivirus strain v810 (16) showed cross-reactivity with the Hom-1 capsid protein in Western blot and immunofluorescence assays (Fig. 1B and C). Western blot analysis of the infected Vero cell lysates collected at different time points postinfection revealed the synthesis and accumulation of a protein (approximately 61 to 62 kDa) corresponding to virus mature capsid protein VP1, starting between 3 and 6 hpi (Fig. 1B). By using the same antibodies for immunofluorescent staining of Vero cells infected at a low MOI (~100 PFU/3 × 10^6^ cells) at 48 hpi, synthesis of the virus capsid was localized predominantly in the cell cytoplasm (Fig. 1C). Electron microscopy (EM) of the virus collected from the Vero cell growth medium and purified by isopycnic centrifugation demonstrated the presence of 35- to 37-nm virions with cup-like depressions on their surface (data not shown).

Several available human cell lines were screened for the growth of Hom-1. The virus was found to replicate in cells of hepatic origin, HepG2 and HuH7, and in cells derived from transformed colonic epithelial cells, SK-CO15 (17), although with different efficiencies (Fig. 1A and B). When virus growth kinetics in human and Vero cells were compared, human cells showed lower rates of virus replication. Accordingly, under a single-step growth condition of infection (MOI = 5), the maximum production of virus in the infected human cells was observed at 24 to 27 hpi, 6 to 9 h later than in Vero cells, in which virus the titer plateaued after 18 hpi (Fig. 1A). While levels of virus amplification in HuH7 cells could reach those in Vero cells (Fig. 1A), the peak virus titers in HepG2 and SK-CO15 cells were lower by 1.8 and 1.1 log_10_ PFU/ml, respectively. Virus growth was not observed in HeLa cells (Fig. 1C; data not shown).

**FIG 2:**
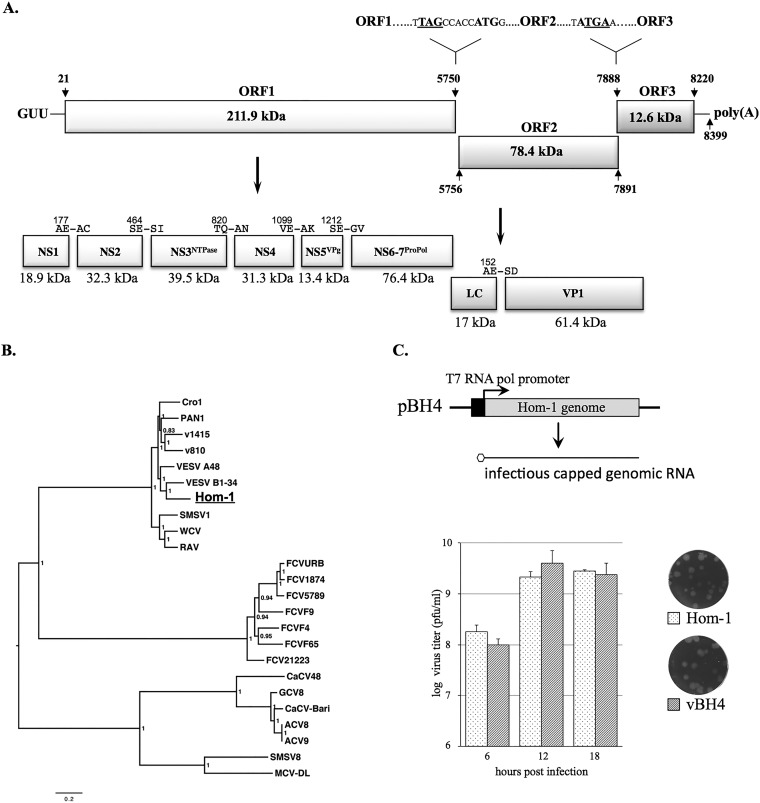
Genetic characterization of the Hom-1 genome. The full-length Hom-1 genome sequence was determined by sequencing overlapping PCR-amplified cDNA fragments. The 5′ and 3′ ends were determined by using RACE kit protocols. (A) Schematic diagram of Hom-1 genome organization. The 8,399-nt virus genome was composed of three ORFs. ORF1 encoded a 211.9-kDa nonstructural polyprotein, ORF2 encoded a 78.4-kDa precursor of virus capsid protein VP1, and ORF3 encoded 12.6-kDa minor capsid protein VP2. The 5′- and 3′-end noncoding regions were 20 and 179 nt in length, respectively. The extreme 5′ end of the Hom-1 RNA genome contained the trinucleotide GUU found only in the CaCV genome. The proteolytic cleavage map of the Hom-1 ORF1 polyprotein and cleavage site in the capsid precursor protein were predicted on the basis of sequence alignment with other vesiviruses and a previously published map of FCV (4, 18). Proteolytic products corresponding to mature virus proteins are depicted as rectangular boxes with calculated molecular sizes shown below and predicted cleavage sites shown above. (B) Phylogenetic relationship of Hom-1 with other vesiviruses. Multiple sequence alignments were produced with MacVector 14.5.3 and Mega 6.06 software for Hom-1 and the following 23 full-length vesivirus genome sequences available in the GenBank database: Reptile calicivirus (Cro1), JX047864; Primate calicivirus 1 (Pan1), AF091736; Steller sea lion vesivirus strain v810 (v810), EF193004; Steller sea lion vesivirus strain v1415 (v1415), EF195384; VESV (VESV A48), U76874; VESV (VESV B1-34), KM269481; the SMSV-1 strain (SMSV1), AF181081; Walrus calicivirus (WCV), AF321298; Rabbit vesivirus (RaV), AJ866991; the FCV Urbana strain (FCVURB), L40021; FCV strain 1874 (FCV1874), JX519214; FCV strain 5789 (FCV5789), JX519210; the FCV F9 strain (FCVF9), M86379; the FCV F4 strain (FCVF4), D31836; the FCV F65 strain (FCVF65), AF109465; FCV strain 21223 (FCV21223), JX519212; CaCV (CaCV48), AB070225; calicivirus isolate Geel 2008/Belgium (GCV8), GQ475303; canine vesivirus Bari/212/07/ITA (CaCV-Bari), JN204722; calicivirus isolate Allston 2008/United States (ACV8), GQ475302; calicivirus isolate Allston 2009/United States (ACV9), GQ475301; the SMSV-8 strain (SMSV8), KM244552; Mink calicivirus MCV-DL/2007/CN (MCV-DL), JX847605. A phylogenetic tree for the alignment of the vesivirus full-length genomes was inferred by the Bayesian method (MrBayes 3.1.2). Bayesian clade probability values are shown next to the nodes. (C) Establishment of a reverse genetics system for Hom-1. A full-length cDNA copy of the Hom-1 genome was assembled in the pX12ΔT (19) vector under the control of the T7 RNA polymerase promoter. The resulting clone, pBH4, was used as a template for the generation of capped genomic RNA in Vero cells infected with MVA/T7, a recombinant vaccinia virus expressing T7 RNA polymerase (20). Intracellular transcription of the Hom-1 RNA led to virus replication and release of viable virus particles. The virus recovered (vBH4) was amplified and compared to the wild-type virus for growth characteristics in Vero cells. Cell monolayers in six-well plates (3 × 10^6^ to 4 × 10^6^/well) were infected with Hom-1 and vBH4 at an MOI of 5, and cell lysates and growth medium were collected at 6, 12, and 18 hpi. Virus titers were determined with a plaque-forming assay. The data represent the mean titers and standard error for each time point.

The Hom-1 genome (8,399 nucleotides [nt] in length) was composed of three ORFs, ORF1 (nt 21 to 5750), ORF2 (nt 5756 to 7891), and ORF3 (nt 7888 to 8220), flanked by short 5′-end (20-nt) and 3′-end (179-nt) noncoding regions (Fig. 2A). While the ORF1 and ORF2 sequences were separated by 5 nt, there was a 4-nt overlap at the ORF2-ORF3 junction, similar to other vesiviruses. Of interest, the extreme 5′ end of the Hom-1 genome contained the trinucleotide sequence GUU (Fig. 2A), which differs from the GUG/GUA sequences found in all other caliciviruses except CaCV (which also has GUU). ORF1 was predicted to encode a 211.9-kDa nonstructural polyprotein. Alignment of the Hom-1 ORF1 polyprotein sequence with that of FCV, which has an experimentally established cleavage map (18), allowed the prediction of five putative cleavage sites and, correspondingly, six mature nonstructural proteins with masses of 18.9 kDa for NS1, 32.3 kDa for NS2, 39.5 kDa for NS3^NTPase^, 31.3 kDa for NS4, 13.4 kDa for NS5^VPg^, and 76.4 kDa for NS6-7^Pro-Pol^ (Fig. 2A). ORF2 would encode a 78.4-kDa precursor of the virus capsid protein, and ORF3 would encode a minor structural protein, VP2, of 12.6 kDa (Fig. 2A). Phylogenetic analysis showed that the Hom-1 virus clustered within the “marine” strains of the genus *Vesivirus* (Fig. 2B), with overall nucleotide sequence identities of those with complete genomic sequences ranging from 75 to 80%. The sequence variability with nonmarine vesiviruses was 49 to 51%. Comparison of the Hom-1 sequence with the partial sequence (~2,000 nt) of the putative parental SMSV-5 strain available in GenBank (GenBank accession no. U18477, U52093, DQ300285, U76884, and U18731) showed only 1% nucleotide sequence differences. Of interest, the sequence of the Hom-1 capsid protein differed from the SMSV-5 sequence by 8 amino acids (aa), with 6 and 2 of the mutations found in the P2 and P1 subdomains, respectively (see Fig. S1 in the supplemental material).

10.1128/mBio.00031-17.1 FIG S1  Hom-1 and SMSV-5 VP1 amino acid sequence alignment. An amino acid sequence of Hom-1 VP1 (aa 263 to 476) was aligned by using MacVector 14.5.3 (MacVector, Inc.) with the corresponding sequence of SMSV-5 VP1 available from GenBank (accession no. U76884). Differences between two sequences are indicated with gray shadowboxes. The borders of the P1 and P2 subdomains (7) are depicted with arrows. Download FIG S1, PDF file, 0.6 MB. Copyright © 2017 Sosnovtsev et al. 2017 Sosnovtsev et al. This content is distributed under the terms of the Creative Commons Attribution 4.0 International license.

The consensus full-length Hom-1 genome sequence was cloned and assembled downstream of the T7 RNA polymerase promoter in the pX12ΔT cloning vector (19) (Fig. 2C). Transfection of the constructed full-length clone, designated pBH4, into Vero cells infected with MVA/T7, an attenuated vaccinia virus expressing the T7 RNA polymerase (20), led to the production of infectious progeny. The recovered virus had growth characteristics similar to those of the wild-type virus (Fig. 2C), and reverse transcription-PCR sequencing of the virus genome confirmed the recovered consensus sequence. In addition, the recovered virus retained the ability to infect human cells, indicating that the consensus capsid sequence recognized its cognate receptor on permissive cells.

### Hom-1 VLPs bind to hJAM1 on the cell surface.

**FIG 3:**
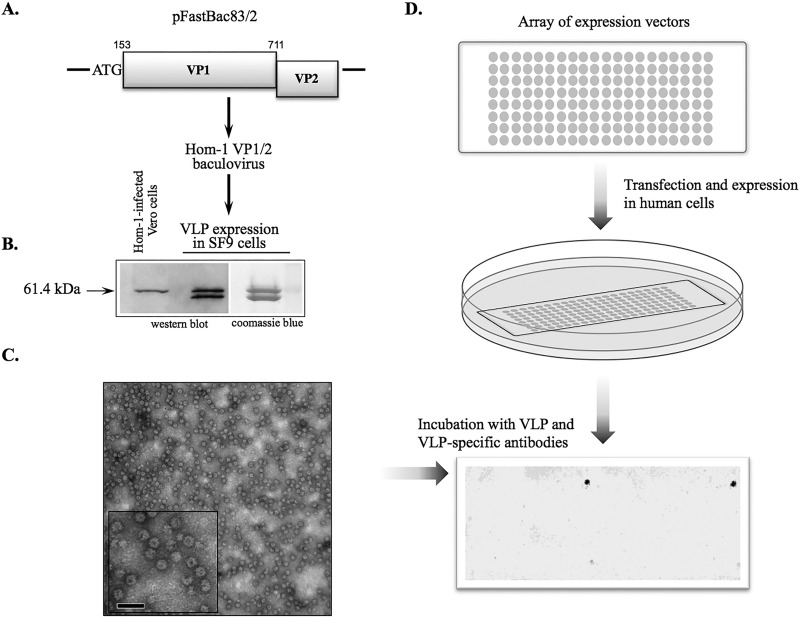
Identification of hJAM1 protein as a binding partner for Hom-1 particles. (A) Recombinant baculovirus expressing Hom-1 VP1 was constructed by recombining a transfer vector (pFastBac83/2) carrying VP1 and VP2 sequences into a bacmid by using Bac-to-Bac technology (Thermo Fischer Scientific, Inc.). An ATG codon was inserted at the beginning of the predicted VP1 gene during PCR cloning of the Hom-1 sequence. (B) Comparison of Hom-1 VP1 synthesized in Vero cells and in insect cells. Vero cells infected with Hom-1 at an MOI of 5 were collected at 8 hpi. After the cells were lysed, the lysate proteins were resolved by SDS-PAGE in a 4 to 20% polyacrylamide gel (Thermo Fischer Scientific, Inc.) and transferred onto a nitrocellulose membrane. The protein (0.6 µg) prepared from VLPs purified with a CsCl gradient from Sf9 cells infected with Hom-1 VP1/VP2 baculovirus was analyzed along with lysates of the infected Vero cells. The membrane was probed with anti-810VLP serum. (C) EM of negatively stained Hom-1 VLPs. The scale bar corresponds to 100 nm. (D) Schematic of VLP-binding screening of the hPMP expression library. Expression vectors encoding 3,559 full-length hPMPs and ZsGreen were arrayed on microarray slides and used for reverse transfection of human cells. Cells expressing hPMPs were treated with VLPs, and bound VLPs were detected with guinea pig anti-VLP serum and fluorescent anti-guinea pig antibodies.

To identify possible receptors involved in the recognition of the Hom-1 virus by human cells, we employed Retrogenix cell microarray screening technology (Retrogenix Ltd., High Peak, United Kingdom). Given that noninfectious VLPs, which are empty virus capsids, would alleviate problems associated with restrictions required in handling the virus, we developed Hom-1 recombinant VLPs. Alignment of the Hom-1 ORF2-encoded protein sequence with that of FCV allowed the prediction of a cleavage site between the Hom-1 LC and mature VP1 at aa 152 and 153 (Fig. 2A). The sequence corresponding to aa 153 to 711 of the Hom-1 ORF2 and all of ORF3 was engineered for expression in the baculovirus system (Fig. 3A), and the resulting recombinant protein of approximately 61 kDa was identical in size to virus VP1 produced in infected cells (Fig. 3B). VP1 expression led to the self-assembly and production of VLPs (Fig. 3C) morphologically and antigenically similar to Hom-1 virions (15). Of note, in some cell lysate and VLP preparations, we detected an additional protein band of approximately 60 kDa (Fig. 3B and data not shown), likely a product of proteolytic degradation by a baculovirus-associated protease. The identity of this smaller protein as a Hom-1 VP1 derivative was confirmed by mass spectrometry analysis (data not shown).

Hom-1 VLPs and anti-VLP serum were used to examine the binding of VP1 to 3,559 distinct hPMPs (Fig. 3D). The corresponding expression vectors, each encoding a full-length version of the membrane protein, were reverse transfected into human cells. Expression of the ZsGreen1 protein, encoded by the same vector, was used as a transfection control. When transfection efficiencies were confirmed to exceed the minimum threshold, Hom-1 VLPs were added at a concentration of 5,000/cell. Screening of the microarrayed, transfected cells for bound VLPs with anti-VLP antibodies identified a single, specific hit that corresponded to the human junctional adhesion molecule 1 (hJAM1) protein. The identity of the protein was confirmed by sequencing of the respective vector.

**FIG 4:**
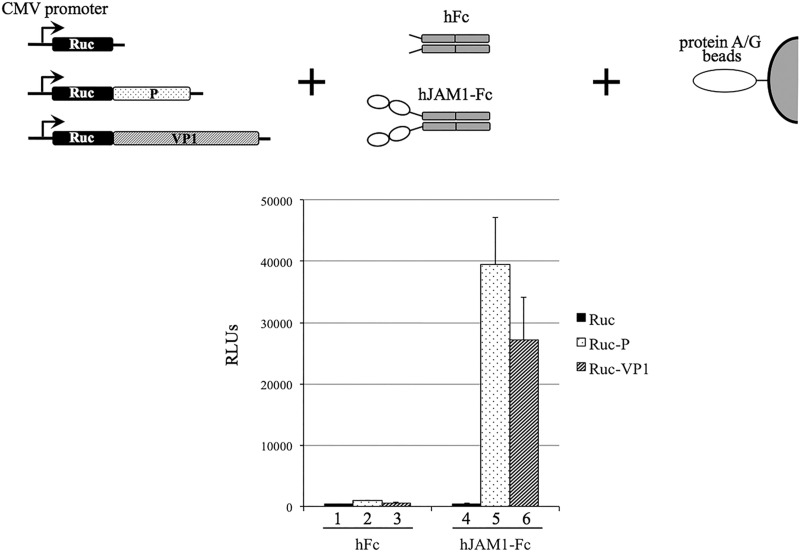
Binding of purified hJAM1-Fc protein to Hom-1 VP1 and the VP1 P domain by LAPD. Lysates of CHO cells expressing Hom-1 VP1 and the VP1 P domain fused to *Renilla* luciferase (Ruc) were prepared at 72 h posttransfection with the corresponding expression vectors as described in Materials and Methods. CHO cells expressing Ruc alone served as a negative control. Equal amounts of RLU were incubated with recombinant hJAM1-Fc or with control hFc protein. The complexes formed were pulled down with protein A/G beads, and following washing, luciferase activity was measured with the coelenterazine substrate. Data represent the mean and standard error of RLU determined from two replicate experiments. CMV, cytomegalovirus.

JAM1 proteins are evolutionarily conserved, and related genes have been found as early as in genomes of protochordates (21). Among mammalian species, the level of protein sequence similarity ranges between 76 and 99%. JAM1 proteins are expressed in a number of tissues, including epithelial, endothelial, and hematopoietic lineage cells. In epithelial and endothelial cells, these proteins are localized to tight junctions, multicomponent structures involved in the formation of intercellular barriers and the control of paracellular flux. The JAM1 proteins belong to the immunoglobulin (Ig)-like protein superfamily and consist of two extracellular Ig-like domains, a transmembrane region, and a short cytoplasmic C-terminal domain (22–24). The ectodomains are involved in homophilic interactions of protein monomers that facilitate dimerization. They also serve as ligands for circulating leukocytes and are implicated in the binding of reovirus and FCV to cells (25, 26). To verify hJAM1–Hom-1 interactions, we employed a luciferase assay for protein detection (LAPD) adapted to examine protein-protein binding (27). The *Renilla* luciferase alone (Ruc) and its fusions to full-length Hom-1 VP1 (Ruc-VP1) or the VP1 P domain (Ruc-P) were expressed in CHO cells, and corresponding cell lysates were prepared as described previously (28). While the luciferase and its fusion proteins served as binding reporters, their targets included recombinant human IgG Fc protein alone (hFc) or hFc fused to the extracellular domain of hJAM1 (hJAM1-Fc). Complexes formed between the Ruc protein and its targets were pulled down with protein A/G beads, and the activity of the Ruc protein was measured with luciferase assay substrate (Promega). Testing of each Ruc fusion protein against both targets revealed the formation of complexes only between hJAM1-Fc and Ruc-VP1 or Ruc-P (Fig. 4). In addition, only background level binding was observed for interactions with hFc (Fig. 4). Similar results were obtained when Ruc fusion proteins were expressed in Cos7 cells (data not shown).

### Expression of hJAM1 confers virus susceptibility to nonpermissive CHO cells.

**FIG 5:**
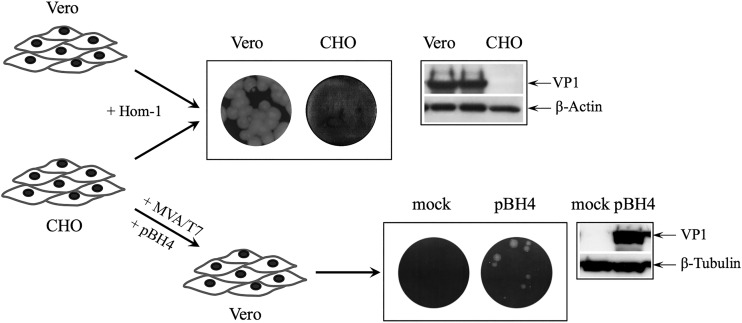
Analysis of Hom-1 replication in CHO cells. Monolayers of CHO and Vero cells were inoculated with Hom-1 by using different dilutions of virus stock. After a 1-h incubation with virus, cells were washed and loaded with complete growth medium containing 1% agar. Monitoring of cell monolayers for several days revealed that no plaques were produced when Hom-1 was inoculated into CHO cells. Lack of virus replication was confirmed by Western blotting of the inoculated CHO cell lysates and later by virus titration analyses and immunofluorescent staining of the cells (Fig. 6). CHO cells supported limited Hom-1 replication and produced infectious virus (~10^2^ PFU/ml) when they were infected with recombinant vaccinia virus prior to being transfected with the infectious full-length cDNA clone of Hom-1 (pBH4).

CHO cells have been described as lacking JAM1 expression (29), and we were unable to infect these cells, even with a high MOI of Hom-1 (Fig. 5). To determine if CHO cells could support Hom-1 replication when an entry barrier was bypassed, approximately 3 × 10^6^ CHO cells infected with MVA/T7 virus were transfected with Hom-1 full-length cDNA clone pBH4, and recovery of infectious virus was assayed in a monolayer of permissive Vero cells. Transfected CHO cells produced viable virus progeny (~10^2^ PFU/ml) that retained parental virus growth characteristics and could reinfect permissive Vero cells (Fig. 5). These data were consistent with the ability of Hom-1 to replicate by using the host machinery of CHO cells and suggested that failure of the virus to grow in these cells was related to the inability of the virus to enter these cells. We next transfected CHO cells with the transient expression vector pC70 (a gift from J. Parker, Cornell University), which contained the entire hJAM1 ORF under the control of a cytomegalovirus promoter. In a majority of the transfected cells expressing hJAM1, the protein exhibited a surface localization pattern. Inoculation of transfected cells with the Hom-1 virus, followed by cell fixation and staining at 18 hpi with antibodies specific for hJAM1 and Hom-1 VP1, revealed efficient synthesis of the virus capsid protein in cells expressing the recombinant protein (data not shown). However, the assessment of virus replication in these cells was obscured by toxicity due to transfection and overexpression of the recombinant protein.

**FIG 6:**
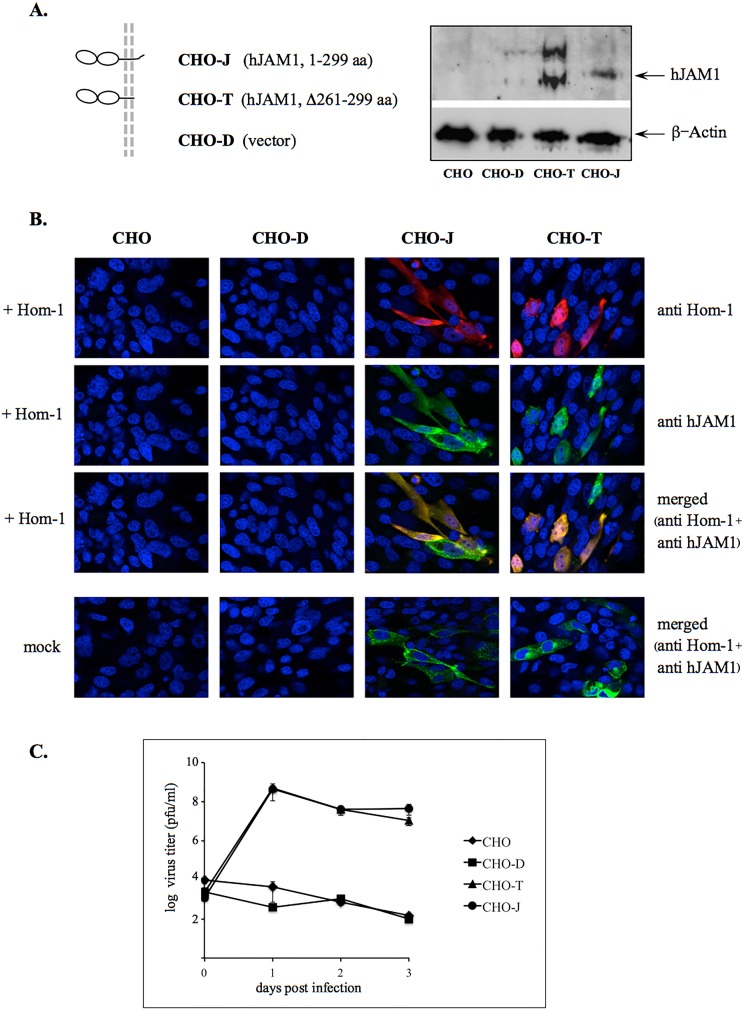
CHO cells stably expressing hJAM1 support Hom-1 replication. (A) Cells stably expressing full-length hJAM1 (CHO-J) or hJAM1 with the C-terminal sequence deleted (Δ261-299 aa; CHO-T), as well as cells transfected with an empty expression vector (CHO-D), were a gift from T. Dermody. Expression of hJAM1 derivatives in these cells was confirmed by Western blotting with anti-hJAM1 antibodies (Acris Antibodies). (B) Immunofluorescent staining of CHO, CHO-D, CHO-J, and CHO-T cells infected with Hom-1. Cells were mock infected or inoculated with Hom-1 at an MOI of 1, incubated at 37°C for 16 h, and fixed with 4% PFA–0.1% Triton X-100 before being stained with guinea pig anti-Hom-1 VLP and mouse anti-hJAM1 (BV16) antibodies. The bound antibodies were visualized with goat anti-mouse IgG and anti-guinea pig IgG antibodies labeled with the Alexa Fluor 488 and 594 dyes, respectively. (C) Growth curve of Hom-1 in stably transfected CHO cells. CHO cells (*n* = 10^7^) were inoculated with Hom-1 at an MOI of 1. After 1 h of incubation at 37°C, the cells were washed to remove unadsorbed virus. Next, growth medium was added and the cells were maintained at 37°C for various times. Infected cells were then collected with growth medium, and virus titers in Vero cells were determined with a plaque-forming assay. The data represent the mean titers of two replicate experiments with the standard error shown for each point.

**FIG 7:**
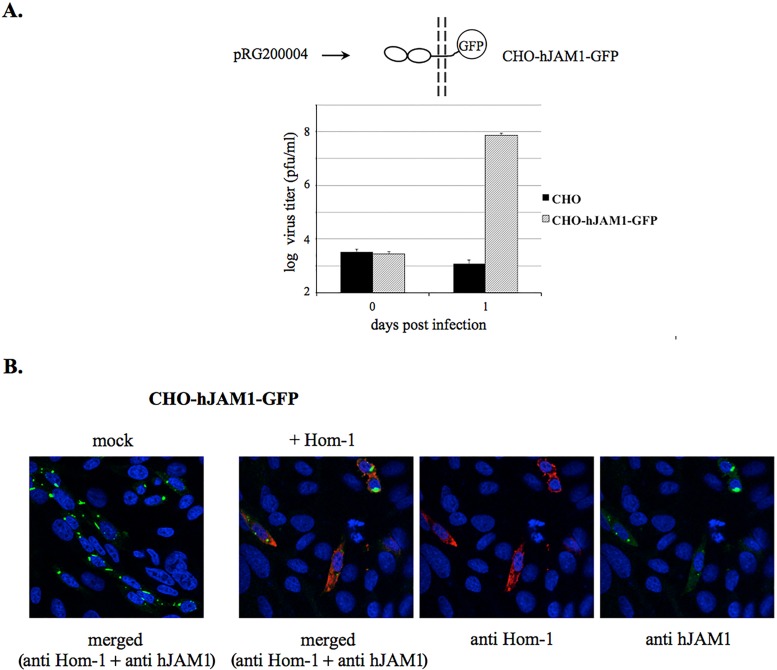
Hom-1 replication in CHO cells expressing hJAM1 fused with fluorescent protein. (A) Growth of Hom-1 in CHO cells transfected with the pRG200004 vector (OriGene Technologies, Inc.) expressing the recombinant hJAM1 protein fused to turboGFP. Transfected CHO cells (CHO-hJAM1-GFP) were grown and expanded in selective growth medium containing G418 at a concentration of 1 mg/ml. Selected cells (*n* = 1.2 × 10^6^) were infected with Hom-1 at an MOI of 1, and cell lysates and growth medium were collected at 1 day postinfection. Virus titers were determined with a plaque-forming assay. The data represent results from two independent experiments with the mean titers and standard error shown for each sample. (B) Immunofluorescent staining of pRG200004-transfected CHO cells that were mock infected or infected with Hom-1 at an MOI of 1. At 12 hpi, infected cells were fixed and stained as described in the Fig. 6 legend.

To improve the efficiency of virus replication, we investigated the use of CHO cells stably expressing hJAM1, designated CHO-J here (Fig. 6A) (29). Inoculation of CHO-J cells with the Hom-1 virus led to successful infection and the production of infectious virus particles (Fig. 6B and C). Similar to transiently transfected cells, staining with antibodies specific for hJAM1 and Hom-1 VP1 confirmed the colocalization of the virus antigen in cells expressing hJAM1 (Fig. 6B). In contrast, control CHO cells, as well as CHO cells transfected with the empty expression vector, displayed no evidence of infection (Fig. 6B). To examine if Hom-1 infection was dependent on the presence of the cytoplasmic tail of the hJAM1 protein, we tested CHO cells stably expressing a 261- to 299-aa deletion mutant form of hJAM1 (29), designated CHO-T here, for virus replication. Similar to that of wild-type hJAM1 protein, expression of the mutant protein with a deleted cytoplasmic domain in nonpermissive CHO cells conferred susceptibility to virus infection (Fig. 6B and C). Titration experiments demonstrated that Hom-1 infection produced similar virus titers in both types of stably expressing cells (Fig. 6C). It is likely that the cytoplasmic part of this protein and, respectively, the C-terminal PDZ domain binding motif (PBM) were not required for efficient virus entry. Supporting the latter conclusion, expression of the C-terminal fusion of the hJAM1 protein and turboGFP (pRG200004; OriGene Technologies, Inc., Rockville, MD) (Fig. 7A) also led to productive infection of CHO cells transfected with the corresponding expression construct (Fig. 7A and B).

### Blocking of hJAM1 with antibodies and genetic knockdown of hJAM1 inhibit Hom-1 replication.

**FIG 8:**
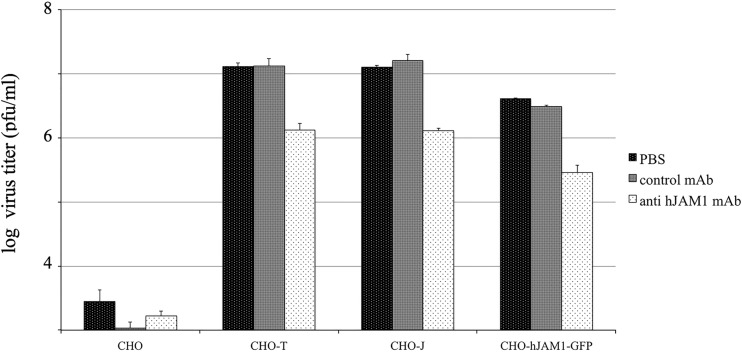
Anti-hJAM1 antibodies reduce Hom-1 infection. CHO, CHO-T, CHO-J, and CHO-hJAM1-GFP cells (*n* = 10^6^) were pretreated with PBS, isotypic control MAbs, or BV16, a MAb specific for hJAM1. After pretreatment, Hom-1 was added to the cells at an MOI of 0.1 and cells were incubated for 1 h before replacement of virus-BV16 inocula with growth medium. At 15 hpi, the infected cells and growth medium were collected. The collected samples were subjected twice to a freeze-thawing procedure and virus titers in Vero cells were measured with plaque-forming assay.

We determined next whether antibodies specific for hJAM1 could block the binding of Hom-1 virus to CHO cells expressing hJAM1. For this purpose, cells were treated with nonrelated or hJAM1-specific antibodies (BV16) or phosphate-buffered saline (PBS) before they were inoculated with a low dose of Hom-1. Analysis of virus replication at early time points postinfection showed that virus titers were lower in cells pretreated with anti-hJAM1 antibodies. At 15 hpi, the levels of inhibition were observed within the range of 84 to 94% (Fig. 8). As expected, PBS and nonspecific control antibodies did not have an inhibitory effect (Fig. 8), suggesting that the titer reduction was a result of blocking of hJAM1 by the BV16 antibodies.

**FIG 9:**
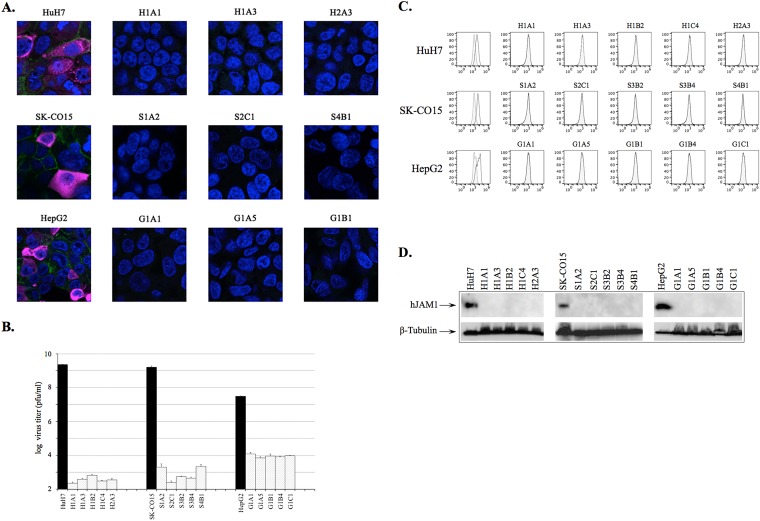
CRISPR/Cas9-mediated knockdown of hJAM1 expression inhibits Hom-1 replication in permissive cells. (A) Immunostaining of selected cloned cell lines with CRISPR/Cas9-edited hJAM1 genes. Cells were grown on glass coverslips in 35-mm-diameter dishes before being mock infected or infected with Hom-1. At 18 hpi, they were fixed and processed as described in Materials and Methods. Staining included DAPI for nuclei (blue), Alexa Fluor 488 for hJAM1 (green), and Alexa Fluor 647 for Hom-1 (purple). Infected-cell images with merged channels are shown. (B) CRISPR/Cas9-modified cells do not support Hom-1 replication. Cells (*n* = 1.5 × 10^6^) were infected with Hom-1 at an MOI of 1. After 1 h of incubation, the inoculum was removed, infected cells were washed, and growth medium was added. Cells were incubated for 24 h at 37°C before being collected. Infected cells were collected with growth medium and freeze-thawed twice, and virus titers in Vero cells were determined with a plaque-forming assay. Black or dotted columns correspond to virus titers in parental or CRSIPR/Cas9-edited cells, respectively. (C) Flow cytometry analysis of hJAM1 expression on the surface of CRISPR/Cas9-edited cells. For flow cytometry, HuH7, HepG2, and SK-CO15 cells and their derivatives were stained with either anti-hJAM1 antibody (black line) or isotypic control MAbs (gray line) conjugated with FITC as described in Materials and Methods. Unstained cells were used as a negative control (shaded gray area). (D) Western blot analysis of hJAM1 expression. For Western blot analysis, cell lysate proteins were resolved in 4 to 10% polyacrylamide gel, transferred onto a nitrocellulose membrane, and probed with anti-hJAM1 antibodies (Acris Antibodies).

To investigate whether loss of hJAM1 expression would affect the susceptibility of permissive cells of human origin (HuH7, HepG2, and SK-CO15) to Hom-1 infection, we attempted to reduce the expression of the corresponding gene by CRISPR/Cas9-mediated mutagenesis. First, Western blot analysis and immunofluorescent staining confirmed the expression of hJAM1 in the three cell lines (Fig. 9A and D; see Fig. S2). Consistent with previously published data (30, 31), the HepG2 and SK-CO15 cells predominantly showed an intercellular distribution of the protein, consistent with localization to tight junctions. However, HuH7 cells expressed less hJAM1, and although the protein was localized to the plasma membrane, in some cells, it displayed an irregular and spotty pattern of distribution (Fig. S2). In an attempt to knock down the expression of the hJAM1 gene, we employed the CRISPR/Cas9 system developed by Santa Cruz Biotechnology, Inc. The protocol involved the transfection of cells with three plasmids that encoded both the Cas9 enzyme and guiding RNA specific to three sites in the hJAM1 gene. The transcribed RNAs would guide Cas9 to its targeted sites to generate double-strand breaks, which were then repaired by homologous recombination. Homology-directed repair (HDR) templates were provided with the set of three HDR plasmids. An advantage of this system was that the directed homologous recombination could insert sequences of red fluorescent protein (RFP) and puromycin *N*-acetyltransferase (PAC). The presence of these markers allowed the selection of transfected cells with puromycin and, after expansion, enrichment of the population of cells with the edited genome by fluorescence-assisted cell sorting (FACS) in the RFP channel. An increase in the proportion of fluorescent cells was observed after each step in the selection process. Figure S2 (see also Fig. S3) illustrates a gradual increase in CRISPR/Cas9-edited HuH7 cells. Insertion of the selectable markers was associated with a corresponding loss of hJAM1 protein expression on the cells’ surface. FACS analysis of the selected cell populations showed low, if any, binding of anti-hJAM1 antibodies conjugated with fluorescein isothiocyanate (FITC) (Fig. S3). In addition, a significant drop in hJAM1 expression was confirmed by Western blot analysis of the proteins from total cell lysates (Fig. S3).

10.1128/mBio.00031-17.2 FIG S2  Immunofluorescent staining of HuH7, SK-CO15, and HepG2 cells infected with Hom-1. Cells were grown on glass coverslips in 35-mm-diameter dishes before being mock infected or infected with Hom-1. At 18 hpi, they were fixed and processed as described in Materials and Methods. Staining included DAPI for nuclei (blue), Alexa Fluor 488 for hJAM1 (green), and Alexa Fluor 594 for Hom-1 (red). The panels on the left show mock-infected cells, and the panels on the right show Hom-1-infected cell images. Download FIG S2, PDF file, 2.9 MB. Copyright © 2017 Sosnovtsev et al. 2017 Sosnovtsev et al. This content is distributed under the terms of the Creative Commons Attribution 4.0 International license.

10.1128/mBio.00031-17.3 FIG S3  (A) Schematic diagram of the CRISPR/Cas9 editing of the hJAM1 gene and selection of cells with the inserted RFP sequence. Cells were transfected with six plasmids, three CRISPR/Cas9 knockout plasmids encoding hJAM1 guide RNAs and three HDR plasmids providing DNA templates for homologous repair with PAC and RFP gene inserts (both sets of plasmids were from Santa Cruz Biotechnology, Inc.). The presence of PAC and RFP genes allowed first selection of the CRISPR/Cas9-edited cells with puromycin (CR cells) and then FACS-mediated enrichment of selected cell populations by using RFP fluorescence (Enr cells). Additional selective pressure was applied when cells were grown in the presence of Hom-1 virus (pH1 cells). Images of cells expressing RFP were collected with a Leica DMI4000B microscope (Leica Microsystems, Inc.) and a Retiga 2000R camera (QImaging). (B) CRISPR/Cas9-mediated editing of the hJAM1 gene reduces Hom-1 replication in HuH7 cells. HuH7, HuH7-CR, HuH7-Enr, and HuH7-pH1 cells (*n* = 1.5 × 10^6^) were infected with Hom-1 at an MOI of 1. After 1 h of incubation, the inoculum was removed, infected cells were washed, and growth medium was added. Cells were either frozen (at the 1-h time point) or incubated for 24 h at 37°C before being collected. Infected cells were collected with growth medium and freeze-thawed twice, and virus titers in Vero cells were determined with a plaque-forming assay. Black or dotted columns correspond to virus titers at 1 or 24 hpi, respectively. (C) Flow cytometry analysis of hJAM1 expression on the CRISPR/Cas9-edited cell surface. For flow cytometry, HuH7, HepG2, and SK-CO15 cells and their derivatives were stained with either anti-hJAM1 antibody (black line) or isotypic control MAbs (gray line) conjugated with FITC as described in Materials and Methods. Unstained cells were used as a negative control (shaded gray area). (D) Western blot analysis of hJAM1 expression. For Western blot analysis, cell lysate proteins were resolved in a 4 to 10% polyacrylamide gel, transferred onto nitrocellulose membrane, and probed with anti-hJAM1 antibodies (Acris Antibodies). Download FIG S3, PDF file, 1.1 MB. Copyright © 2017 Sosnovtsev et al. 2017 Sosnovtsev et al. This content is distributed under the terms of the Creative Commons Attribution 4.0 International license.

Analysis of virus growth in the CRISPR/Cas9-edited cells revealed a considerable reduction in virus replication; a 4-log_10_ drop in the infectivity titers of Hom-1 was observed in the population of selected and bulk-sorted HuH7 cells (Fig. S3). However, immunofluorescence analysis of the infected CRISPR/Cas9-edited cells demonstrated the presence of small amounts of positively stained cells (data not shown). Of interest, one-step incubation of the CRISPR/Cas9-edited cells with the Hom-1 virus led to selection against cells that retained sensitivity to virus infection (Fig. S3). To confirm the direct effects of hJAM1 knockout on virus replication, the selected cells were subjected to FACS-mediated single-cell cloning. The lack of hJAM1 expression in five expanded clones from each cell line was verified by immunostaining, flow cytometry, and Western blot analyses (Fig. 9A, C, and D). When inoculated with Hom-1 virus, cloned cell monolayers showed no CPE and remained negative after immunostaining for Hom-1 capsid antigen (Fig. 9A). Analysis of virus growth confirmed a further reduction of virus titers compared to those of uncloned populations of the CRISPR/Cas9-edited cells (Fig. 9B; see Fig. S3). The genetic identity of the cloned cells to parental cells was confirmed by short tandem repeat profiling (Genetica DNA Laboratories). Altogether, these data suggest that the knockdown of hJAM1 expression makes otherwise permissive cells insensitive to virus infection.

## DISCUSSION

Human JAM1 was identified as a receptor with high affinity for calicivirus strain Hom-1. The receptor is present on human cell lines permissive for Hom-1 virus infection, and CRISPR-mediated knockout of its expression on these cell lines rendered them nonpermissive. Reciprocally, transfection of the nonpermissive CHO cell line with an expression vector providing hJAM1 rendered them permissive for Hom-1 infection. The identification of a functional calicivirus receptor on human cells provides further insight into the entry mechanisms of caliciviruses. Although carbohydrate moieties facilitate virus binding to cells in many caliciviruses (32), including norovirus, the major functional virus receptor(s) is likely to be a cell surface protein (26, 33, 34).

This work was prompted by the description of Hom-1 as an SMSV-like vesivirus that was accidentally transmitted to a laboratory worker (15). Caliciviruses, including noroviruses, have generally exhibited strong species tropism, but the natural host distribution of the marine vesiviruses appeared broad (reviewed in reference 8). Published reports of antibodies and vesivirus sequences in human sera (35–37) suggested zoonotic transmission to humans, and it was important to explore whether the marine vesiviruses could infect human cells. Our approach initially involved the screening of our collection of human and animal cell lines for productive infection by Hom-1. In our previous experience with FCV, permissive cell lines of human origin were not found and the cell lines that were permissive were nearly all of feline origin. However, many nonpermissive cell types (including those of human origin) could support productive FCV infection when transfected with infectious FCV RNA or when expressing FCV RNA transcribed intracellularly from transfected infectious cDNA clones (38). We anticipated a similar pattern with the Hom-1 vesivirus but instead found that the human cell lines HuH7, SK-CO15, and HepG2 were fully permissive for Hom-1 infection. These results prompted the screening of a hPMP expression library for binding to Hom-1 VLPs, and hJAM1 was identified as a functional receptor for Hom-1 on permissive human cells.

The hJAM1 protein is related to fJAM1, which was previously identified as a receptor for another vesivirus, FCV (26, 39, 40). They both belong to a group of structurally conserved membrane proteins expressed by multiple cell types, including epithelial and endothelial cells, and cells of hematopoietic origin. In epithelial and endothelial cells, JAM1 proteins are usually localized at cell-cell contacts, where they are involved in the assembly and maintenance of tight junctions. Structurally, JAM1 proteins belong to the superfamily of immunoglobulin-like (IgSF) proteins; they contain two concatenated extracellular domains (D1 and D2) with Ig-type folding, a short transmembrane segment, and a cytoplasmic tail that carries a PBM at the C terminus (22–24). Their extracellular domains contain a dimerization R(V,I,L)E motif that mediates the formation of the protein dimers in *cis* (24, 41). Pairs of these JAM1 *cis* dimers from adjacent cells can interact in *trans*, forming a zipper-like structure important for the tight junction barrier function (41). Homodimerization of JAM1 in *cis* is thought to also play a regulatory role in the outside-in signaling mediated by interactions of this protein cytoplasmic domain with several intracellular scaffolding proteins (42). Some of the heterophilic associations of JAM1 have been implicated in the regulation of cell migration (43). Proteins in the IgSF superfamily serve as receptors for a number of viruses. Included among them are rhinovirus (intercellular adhesion molecule 1 or ICAM-1), herpes simplex virus (nectin 1, and 2), measles virus (signaling lymphocyte-activation molecule or SLAM), rabies virus (neural cell adhesion molecule or NCAM-1), coronavirus (carcino-embryonic antigen glycoprotein or CEACAM), and coxsackievirus and adenovirus (coxsackievirus and adenovirus receptor or CAR) (reviewed in references 44 and 45). Although viral IgSF receptors differ in the number and folding type of their Ig-like domains, the domain responsible for binding in the majority of these viruses includes a V-type fold located at the protein N terminus (40, 45). The recently identified receptors for murine norovirus (MNV), CD300lf and CD300ld, also belong to the IgSF group and have single Ig-like domains (33, 34), expanding the range of these molecules as viral receptors.

Recovery of infectious Hom-1 particles from a full-length cDNA clone in CHO cells, otherwise resistant to Hom-1 infection, confirmed the presence of host cell machinery required for virus protein and RNA synthesis and virion assembly. Although recovered virus particles were fully competent in the infection of permissive Vero cells, they could not reinfect CHO cells. In characterizing the role of hJAM1–Hom-1 VP1 interactions in virus entry, the expression of hJAM1 in CHO cells rendered them susceptible to infection. Conversely, treatment of transfected cells with monoclonal antibodies (MAbs) specific for hJAM1 considerably reduced virus replication. These findings were consistent with the hJAM1 molecule having a functional role in Hom-1 recognition and entry and suggested that the rest of the internalization pathway was sufficiently present in CHO cells. However, further testing of cells expressing hJAM variants raised questions about the exact mechanisms involved in the triggering of virus particle internalization. For example, analyses of CHO cells stably expressing the hJAM1 protein lacking its cytoplasmic tail revealed that virus infection could be supported. This observation indicated that virus internalization was not dependent on signaling provided by the cytoplasmic domain of hJAM1. Consistent with that, we found that Hom-1 could efficiently enter cells expressing an hJAM1-green fluorescent protein (GFP) fusion, where the GFP moiety was expected to mask the hJAM1 PBM.

The cytoplasmic domain has been shown to be dispensable for the functional MNV receptor CD300lf (33, 34). In contrast, removal of the cytoplasmic tail from fJAM1 had a negative effect on FCV infection of cells expressing the corresponding truncated protein. While these cells retained virus-binding characteristics, no expression of virus capsid was detected inside, which is indicative of failure of the virus to enter cells (39). The differences between Hom-1 and FCV infections observed in the present study and FCV studies can be explained by the presence or absence of a transmembrane region in the JAM1 mutants tested. The transmembrane region of the fJAM1 mutant was replaced with a glycosyl-phosphatidylinositol anchor of human decay-accelerating factor (39), while the mutant hJAM1 protein in our study had an authentic transmembrane sequence (29). Little is known about the functional role of the JAM1 transmembrane segment; however, it is possible that this part of the receptor molecule plays some signaling role in virus entry. Of interest, the transmembrane domain of CD300d, which is a human ortholog of mouse CD300ld, which has also been implicated in MNV entry, interacts with transmembrane adaptor proteins FcϵRγ and DAP12, which are known to be parts of signal transduction pathways (34, 46). Possible interactions of hJAM1-bound Hom-1 virus particles with other cell surface molecules could offer an alternative pathway for triggering virus internalization. The σ1 protein of the reovirus virion has been reported to engage the JAM1 receptor (25), and, similar to Hom-1, the reovirus could efficiently enter cells expressing JAM1 lacking a cytoplasmic domain (29). The molecule responsible for the enhancement of virus internalization was suggested to be β1 integrin (47). Supporting that, reovirus entry was substantially diminished in β1-deficient, compared to β1-expressing, cells, while binding of the virions to the cells remained at similar levels (47). Mutations in the cytoplasmic part of β1 integrin, encoding endocytotic signals, led to the localization of virus particles in compartments different from endosomes (48). In addition, treatment of cells with antibodies specific to β1 integrin reduced reovirus infectivity (47). Whether Hom-1 employs additional internalization receptors upon entry and how uncoating occurs to release the genomic RNA require further study. However, the engagement of hJAM1 by Hom-1 is likely a key starting point in the virus entry process in human cells. CRISPR/Cas9 inactivation of the hJAM1 gene resulted in a substantial loss of Hom-1 infectivity in all three permissive human cell lines tested.

Our work demonstrates that the Hom-1 vesivirus can infect human cells *in vitro* and that hJAM1 is utilized as the receptor on these cells for virus entry. This finding is consistent with the apparent infection of a laboratory worker following direct exposure to the virus (15). The ability to infect human cells with Hom-1, a virus that groups with the marine vesiviruses, suggests that cross-species transmission is at least feasible, although it does not illuminate the natural history of these viruses. It will be important to distinguish between active infection and passive exposure to nonreplicating antigens in the environment. An important remaining question is whether vesiviruses cause significant human disease. Such a case has been made for the marine vesiviruses in studies that have reported a higher prevalence of vesivirus antibodies in certain patient groups with hepatic disease (36, 37). It is noteworthy that virus discovery efforts by deep sequencing have not detected vesivirus RNA in human clinical samples (49), but vesivirus sequences have been detected with these techniques in sea lions and other wild animals (50). These data suggest that species tropism, largely determined by receptor specificity, is responsible for the host range of these viruses and that interspecies transmission is a rare event. However, the surprising identification of a functional receptor on human cells indicates that some calicivirus receptors can be shared across species and continued epidemiological monitoring of caliciviruses is warranted.

## MATERIALS AND METHODS

### Cells and virus.

Vero, Cos7, HepG2, HeLa, and CHO cells were purchased from the American Type Culture Collection (Manassas, VA) and maintained according to the manufacturer’s instructions. HuH7 cells (a gift from S. Emerson, National Institute of Allergy and Infectious Diseases [NIAID]) and HEK293T cells (a gift from M. Morelli, NIAID) were maintained in Dulbecco modified Eagle medium (DMEM)-GlutaMAX (Thermo Fisher Scientific, Inc., Carlsbad, CA) supplemented with 10% fetal bovine serum (FBS), 100 U of penicillin, and 100 µg/ml streptomycin (1×Pen/Strep; Mediatech, Manassas, VA). CHO-J, CHO-T, CHO-D (gifts from T. Dermody, Vanderbilt University), and CHO-F (a gift from J. Parker, Cornell University) cells were grown in F12-GlutaMAX medium (Thermo Fisher Scientific, Inc.) supplemented with 10% FBS, 1 mg/ml Geneticin (Thermo Fisher Scientific, Inc.), and 1×Pen/Strep (Mediatech). SK-CO15 cells (a gift from E. Rodriguez-Boulan, Cornell University, and A. Ivanov, Virginia Commonwealth University) were maintained in DMEM-GlutaMAX (Thermo Fisher Scientific, Inc.) supplemented with 10% FBS, 15 mM HEPES, 1% nonessential amino acids (Sigma-Aldrich, St. Louis, MO), and 1×Pen/Strep (Mediatech).

To amplify the virus, a confluent monolayer of Vero cells, grown in a 175-cm^2^ flask, was inoculated with 0.2 ml of the original virus stock received from ATCC. The development of a CPE was monitored, and after it exceeded 90%, the growth medium was collected. Supernatant was cleared by low-speed centrifugation, aliquoted, and then stored at −80°C.

To analyze virus replication, single-step and multistep growth curve experiments were carried out with cell monolayers seeded into six-well plates. Cells were inoculated with the Hom-1 virus at an MOI of 5 or 0.05. Following 1 h of incubation at 37°C, the inoculum was removed and the cells were washed with 1 ml of growth medium. After washing, 2 ml of growth medium was added and cells and culture fluids were collected at the postinfection times indicated. The collected samples were subjected to two freeze-thaw cycles, and virus titers in Vero cells were determined with a plaque-forming assay. The titration experiments were carried out similarly to what has been described previously for FCV (51).

### Sequencing and sequence analysis.

Viral RNA purified with the Qiagen RNeasy kit was employed to synthesize cDNA with random hexamer primers. Several cDNA fragments overlapping the entire genome were amplified with multiple pairs of primers (available on request). Following gel purification, direct sequencing of the amplified cDNA fragments was performed with an automated sequencer and a genome walking procedure. The 3′- and 5′-end sequences of the Hom-1 genome were determined with rapid amplification of cDNA ends (RACE) system kits from Roche Applied Science (Indianapolis, IN) or Thermo Fisher Scientific, Inc. Derived nucleotide and amino acid sequences were analyzed, aligned, and compared with sequences available from GenBank with the MacVector 14.5.3 (MacVector, Inc., Apex, NC), Mega 6.06 (52), and MrBayes 3.1.2 (53) software packages.

### Full-length genomic cDNA clone construction and virus recovery.

Standard recombinant DNA methods were used for plasmid construction (54). To assemble the full-length sequence of the Hom-1 virus genome in pX12ΔT (a gift from U. Buchholz, NIAID), the 5′-end (nt 1 to 4068) and 3′-end (nt 4069 to 8399) parts of the virus genome were amplified and cloned separately into the pSPORT1 vector (Thermo Fisher Scientific, Inc.). The 5′-end sequence was amplified from cDNA with primers 5′ ATTTATTTAAT**GGTCTC**AAGCTTTAATACGACTCACTATAGTTAAATGAGAATTTGAGCTATGGCTC 3′ and 5′ CTTGTCACCACCCGATCC 3′. The sequence of the first primer contained 27 nt corresponding to the beginning of the Hom-1 genome (underlined), the T7 RNA polymerase promoter (in italics), and BsaI site (in bold). The second primer contained a sequence complementary to nt 4071 to 4088 of the Hom-1 genome. The amplified cDNA fragment was treated with BsaI and SphI and cloned into HindIII-SphI-linearized pSPORT1. The 3′-end sequence was amplified with primers 5′ TTGTGGGCTACCATACACCGACGAAC 3′ and 5′-AATTTAATAT**CCCGGG**TTTTTTTTTTTTTTTTTTTTTTTTTTTTTTCCTAATGCAACCTACCAATTGGCTAAGTC-3′. The first primer corresponded to nt 4019 to 4044 of the Hom-1 genome, and the second primer contained a sequence complementary to the last 29 nt of the virus genome (underlined), a poly(T_30_) sequence, and an XmaI site (in bold). The amplified cDNA fragment was treated with XmaI and SphI and cloned into XmaI-SphI-linearized pSPORT1. Clones were screened by sequencing analysis, and plasmids containing consensus sequences of the 5′ and 3′ parts of the virus genome were selected and designated p548 and p432, respectively. To construct a full-length genome clone, plasmids p548 and p432 were treated with SmaI and SphI or SphI and XmaI, respectively. The 4,133-bp SmaI-SphI and 4,362-bp SphI-XmaI fragments containing nt 1 to 4068 and 4069 to 8399 of the virus genome, respectively, were purified and ligated into the EcoRV-XmaI-linearized pX12ΔT vector (19). Clones were screened by restriction analysis and selected for further sequencing analysis. The resulting plasmid was designated pBH4 and contained the full-length consensus genome sequence of Hom-1 placed under the control of the T7 RNA polymerase promoter.

Transfection of MVA-T7-infected Vero or CHO cells with pBH4 and virus recovery of Hom-1 were performed as described previously for FCV (38). Recovery of infectious virus was monitored by detection of a CPE in the monolayer of Vero cells and by further virus titration.

### Recombinant baculovirus construction, VLP expression, and anti-VLP serum production.

To express Hom-1 VLPs, the Bac-to-Bac Baculovirus Expression System and *Spodoptera frugiperda* cells (Sf9; Thermo Fisher Scientific, Inc.) were used. Briefly, the 2,006-bp sequence encoding Hom-1 VP1 (aa 153 to 711 of ORF2) and VP2 (ORF3) was amplified with primers HVLPfrw (5′-ATTTATATTA**GGATCC**ACCATGTCGGATGGTCCGGGAAGCTCCGAGATTGTG-3′) and HVLPrev (5′-ATATTTATTTA**GGTACC**TTACTAGTCCGTTTTATAGAAGCTATAATAAGAG-3′). The primer sequences contained BamHI and KpnI restriction enzyme sites (in bold), an ATG translation initiation codon, and a sequence corresponding to two stop codons (underlined). The PCR fragment was treated with BamHI and KpnI and cloned downstream of the polyhedrin promoter into the baculovirus donor plasmid, pFastBac-1 (Thermo Fisher Scientific, Inc.) linearized with the corresponding restriction enzymes. Baculovirus DNA containing the Hom-1 VP1-VP2 sequence was produced by site-specific recombination in *Escherichia coli* DH10Bac cells harboring a bacmid vector according to the manufacturer’s protocol. The resulting baculovirus was isolated from Sf9 cells transfected with the recombinant bacmid DNA with Cellfectin (Thermo Fisher Scientific, Inc.). After three consecutive passages, the amplified baculovirus was titrated, aliquoted, and stored for further experiments. For protein expression, Sf9 cells were infected with baculovirus at an MOI of 5, and at 6 days postinfection, the cells and growth medium were freeze-thawed twice and VLPs were purified as previously described (55). Antisera against Hom-1 VLPs were prepared in guinea pigs as previously described (56). All guinea pig studies were conducted at the NIH, Bethesda, MD, under an animal protocol (LID 73) approved by the NIAID Division of Intramural Research Animal Care and Use Committee.

### Western blot and immunofluorescence analyses.

Western blot analysis of virus and cellular proteins in cell lysates was performed with standard techniques (54). The virus capsid protein was detected with guinea pig polyclonal sera raised against VLPs that were produced either for Steller sea lion vesivirus strain v810 (16) or for the Hom-1 virus. Anti-β-actin and anti-β-tubulin antibodies were obtained from Sigma-Aldrich. Mouse MAb 2E3-1C8 from Acris Antibodies, San Diego, CA, was used for the detection of hJAM1. Bound primary antibodies were detected with horseradish peroxidase-labeled secondary antibodies that were purchased from Kirkegaard & Perry Laboratories, Gaithersburg, MD. Blots were developed with Amersham ECL Western blotting Reagents (GE Healthcare Life Sciences, Buckinghamshire, United Kingdom).

For immunofluorescent staining, cells grown in six-well tissue culture plates were washed with 2 ml of PBS, fixed with 4% paraformaldehyde (PFA) for 10 to 15 min at room temperature, and permeabilized in 0.1% Triton X-100 for 10 min prior to washing with PBS and the addition of primary antibodies diluted in PBS containing 1% normal goat serum (Kirkegaard & Perry Laboratories). After a 1-h incubation, the cells were washed with PBS and binding of the primary antibodies was detected by the addition of affinity-purified Alexa Fluor-labeled goat antibodies (10 µg/ml) raised against either guinea pig or mouse IgG (Thermo Fisher Scientific). For nuclear staining, mock- and Hom-1-infected cells were treated for 20 min with a PBS solution containing 1 µg/ml DAPI (4′,6-diamidino-2-phenylindole; Sigma-Aldrich). Fluorescent staining was visualized with a Leica DMI4000B microscope (Leica Microsystems, Inc., Buffalo Grove, IL). Images were acquired with a Retiga 2000R camera (QImaging, Surrey, BC, Canada) and processed with iVision 4.5.5 software (BioVision, Exton, PA). For confocal microscopy experiments, cells were grown on glass coverslips in 35-mm-diameter dishes before being transfected with plasmid DNA or infected with the virus. The cells were then fixed, stained, and processed as previously described (57). Confocal microscopy images were obtained with a Leica TCS-SP5 confocal microscope (Leica Microsystems, Inc.) equipped with a white laser and with a 63× oil immersion objective. The collected images were analyzed with Imaris software (Bitplane, Zurich, Switzerland).

### Screening of the library of hPMPs.

Screening for human protein ligands of the Hom-1 virus was performed by the Retrogenix Cell microarray technology (Retrogenix Ltd.). For the binding analysis, 3,559 expression vectors, each encoding a full-length hPMP and ZsGreen1 protein (Clontech, Mountain View, CA), were arrayed in duplicate on 10 microarray slides. The vectors were reverse transfected into human HEK-293 cells, and ZsGreen1 levels were measured to ensure that efficient transfection had been achieved. Hom-1 VLPs (5,000/cell) were added to the slides after cell fixation. The slides were incubated at room temperature for 1 h, and VLP binding was detected with anti-Hom-1 VLP hyperimmune guinea pig serum (diluted 1:2,000 in PBS containing 0.5% BSA) and Alexa Fluor 647-labeled goat anti-guinea pig IgG (diluted 1:500 in PBS–0.5% BSA). Slides incubated with both hyperimmune serum and anti-guinea pig IgG antibodies were used as a negative control. Fluorescent images were collected with an Ettan difference gel electrophoresis fluorescence scanner (GE Healthcare Life Sciences) and analyzed with ImageQuant software (GE Healthcare Life Sciences).

### LAPD.

A modified version of the LAPD assay (27) was employed to test interactions of hJAM1 and Hom-1 VP1 *in vitro*. Briefly, CHO cells (*n* = 2.4 × 10^7^) were transfected with expression constructs containing the *Renilla* luciferase (Ruc) gene fused to the Hom-1 VP1 (Ruc-VP1) sequence or its P domain (Ruc-P) sequence (28). Two days after transfection, cells were lysed with 1.4 ml of lysis buffer containing 50 mM Tris (pH 7.5), 100 mM NaCl, 5 mM MgCl_2_, 1% Triton X-100, 50% glycerol, and 1× Halt protease inhibitor cocktail (Thermo Fisher Scientific. Inc.). The lysates were cleared by centrifugation at 12,000 × *g* for 5 min, aliquoted, and stored at −80°C. In addition, lysates of cells transfected with the empty expression vector pRen2 (58) were prepared for use as a negative control. The luciferase activity in the cell lysates obtained was assayed with the *Renilla* luciferase assay substrate (Promega, Madison, WI), and luminescence was measured in relative light units (RLU) with the Synergy Neo2 Multi-Mode Reader (Bio-Tek Instruments, Inc., Winooski, VT). To examine Hom-1 VP1 interactions with hJAM1, cell lysates containing equal amounts of RLU (*n* = 10^7^) were incubated with 0.5 µg of either hJAM1-Fc (Sino Biological Inc., Beijing, China) or hFc (G&P Biosciences, Santa Clara, CA) recombinant protein for 1 h at room temperature. Cell lysate-recombinant protein mixtures were then transferred to a 96-well filter HTS plate (EMD Millipore, Billerica, MA), where each well was preloaded with 5 μl of a 30% suspension of protein A/G Plus UltraLink Resin beads (Thermo Fisher Scientific) in PBS. The filter plate with samples was incubated for 1 h at room temperature on a rotary shaker. The samples were washed seven times on a vacuum manifold (Bio-Rad Laboratories, Hercules, CA) with a buffer containing 50 mM Tris (pH 7.5), 100 mM NaCl, 5 mM MgCl_2_, and 1% Triton X-100 at 200 μl/well and three times with PBS at 100 μl/well. Following washing, the amount of Ruc antigen bound to hJAM1-Fc was determined by measuring luminescence as described above.

### Cell transfection and selection.

For transient expression, cells (2 × 10^6^ to 3 × 10^6^/well) were transfected with 1 to 5 µg of plasmid DNA by using Lipofectamine 2000 or 3000 Reagent (Invitrogen) and the protocols supplied by the manufacturer. Transfected cells were incubated for 24 to 48 h before further manipulations. For transfected cell selection, cells were grown in the presence of the antibiotic G418 (1 mg/ml) or puromycin (2 to 5 µg/ml) (Thermo Fisher Scientific, Inc.).

### MAb-mediated inhibition of virus infection.

To assess the effects of an anti-hJAM1-specific MAb on Hom-1 entry, CHO cells expressing different versions of hJAM1 protein were treated with MAb BV16 (Hycult Biotech Inc., Plymouth Meeting, PA) prior to infection with the virus. Briefly, a commercial preparation of MAb BV16 was dialyzed against PBS to remove traces of sodium azide, and 4 µg of antibodies was added to 10^6^ cells in 200 µl of PBS. After 30 min of incubation, the cells were inoculated with virus at an MOI of 0.1, which was added in 200 µl of growth medium. Inocula were removed after 1 h of incubation, and cells were loaded with 1 ml of growth medium. At 15 hpi, cells and growth medium were collected and subjected to two freeze-thaw cycles and virus titers were determined by plaque-forming assay. Cells pretreated with PBS only or with MAb IgG1 isotype control antibodies (Hycult Biotech Inc.) were used as a negative control.

### CRISPR/Cas9-mediated knockdown of hJAM1.

To knock down the F11R gene, HuH7, HepG2, and SK-CO15 cells were transfected with a mixture of JAM-A CRIPSR/Cas9 knockout and corresponding JAM-A HDR plasmids (Santa Cruz Biotechnology, Inc., Dallas, TX). Plasmid transfections were performed with UltraCruz Transfection Reagent (HuH7 and SK-CO15) or Lipofectamine 3000 (HepG2) in accordance with the protocols provided by the manufacturers (Santa Cruz Biotechnology, Inc., and Thermo Fisher Scientific, Inc., respectively). Efficiency of transfection was monitored by detection of the expression of vector-encoded GFP and RFP by fluorescence microscopy. Transfected cells were selected with medium containing puromycin (5 µg/ml for HuH7 and SK-CO15 cells and 2 µg/ml for HepG2 cells). Expanded pools of cells resistant to puromycin were maintained in the corresponding selective medium. After three or four passages, they were further enriched for cells expressing RFP by bulk sorting on a FACS Aria II (BD Biosciences, San Jose, CA) equipped with 488-, 405-, 561-, and 633-nm lasers and a 100-mm nozzle at a sheath pressure of 20 lb/in^2^. First, live cells were gated by using forward scatter area versus side scatter area. Three sets of gates that included side scatter height versus side scatter width, forward scatter height versus forward scatter width, and forward scatter width versus side scatter area were then used to exclude doublets. Cells were collected in 15-ml tubes with the corresponding growth medium. The same sorter was used to deposit single cells into 96-well plates for clonal selection. The clones obtained were expanded and assayed for hJAM1 expression. The knockdown of this protein was confirmed by flow cytometry and Western blot analyses. For flow cytometry, live cells (*n* = 2 × 10^6^) were stained with FITC-conjugated anti-human CD321 (F11R) antibodies (BioLegend, San Diego, CA) as recommended by the manufacturer and analyzed with a BD Accuri C6 flow cytometer (BD Biosciences, San Jose, CA). Unstained cells and cells stained with FITC-conjugated isotype control antibodies (BioLegend) were used as fluorescent labeling controls.

The authentication of selected clones, as well as of parental cell lines, was conducted at Genetica DNA Laboratories, Burlington, NC.

### Accession number(s).

The genome sequence of Hom-1 was submitted to GenBank and assigned accession no. KY114613.
