# Supplementary material for: Identification of Human Junctional Adhesion Molecule 1 as a Functional Receptor for the Hom-1 Calicivirus on Human Cells
Source: mBio. 2017 Feb 14;8(1):e00031-17. doi: 10.1128/mBio.00031-17 (PMC5312078; doi:10.1128/mBio.00031-17)
Supplement: FIG S1 [file mbo001173190sf1.pdf]

```
        P2
        →
Hom-1   WSTPYYVPIEIKLHAETKANNRWFHVIDTDKALVPGLPDGWPDTTIPESVTATNGDFAYATDFYNPATKTVDPTKNTTPFKGTYICGTLSTVTIPEVDNQNYAKKEA
SMSV-5  WSTPYYVPIEIKLHAETKANNRWFHVIDTDKALVPGLPDGWPDTTIPESVTATNGDFAYATDFYNPATKTVDPTKNTTPFKGTYICGTLSTATIPEVDNQNYAKKEA

                                                                                      P2 P1
                                                                                      ← →
Hom-1   QKKSQTMYITTANIGDGNASPQHKISPQKLIVFFDGPESTMDINVTLCPLGFTLVDGQPIGSSSSKVVRIATLPEAITQGGNYPIFYVNKVKIGYFDRQTTECYNSQ
SMSV-5  QPKSQTMYITTADIGNGNASPQRKISPQKLIVFFDGPESTMDINVTLSPLGFTLVDGQPIGSSSSKVVRITTLPEAITQGGNYPIFYVNKVKVGYFDRQTTECYNSQ
```
