# Supplementary figures and images for: Identification of Human Junctional Adhesion Molecule 1 as a Functional Receptor for the Hom-1 Calicivirus on Human Cells
Source: mBio. 2017 Feb 14;8(1):e00031-17. doi: 10.1128/mBio.00031-17 (PMC5312078; doi:10.1128/mBio.00031-17)

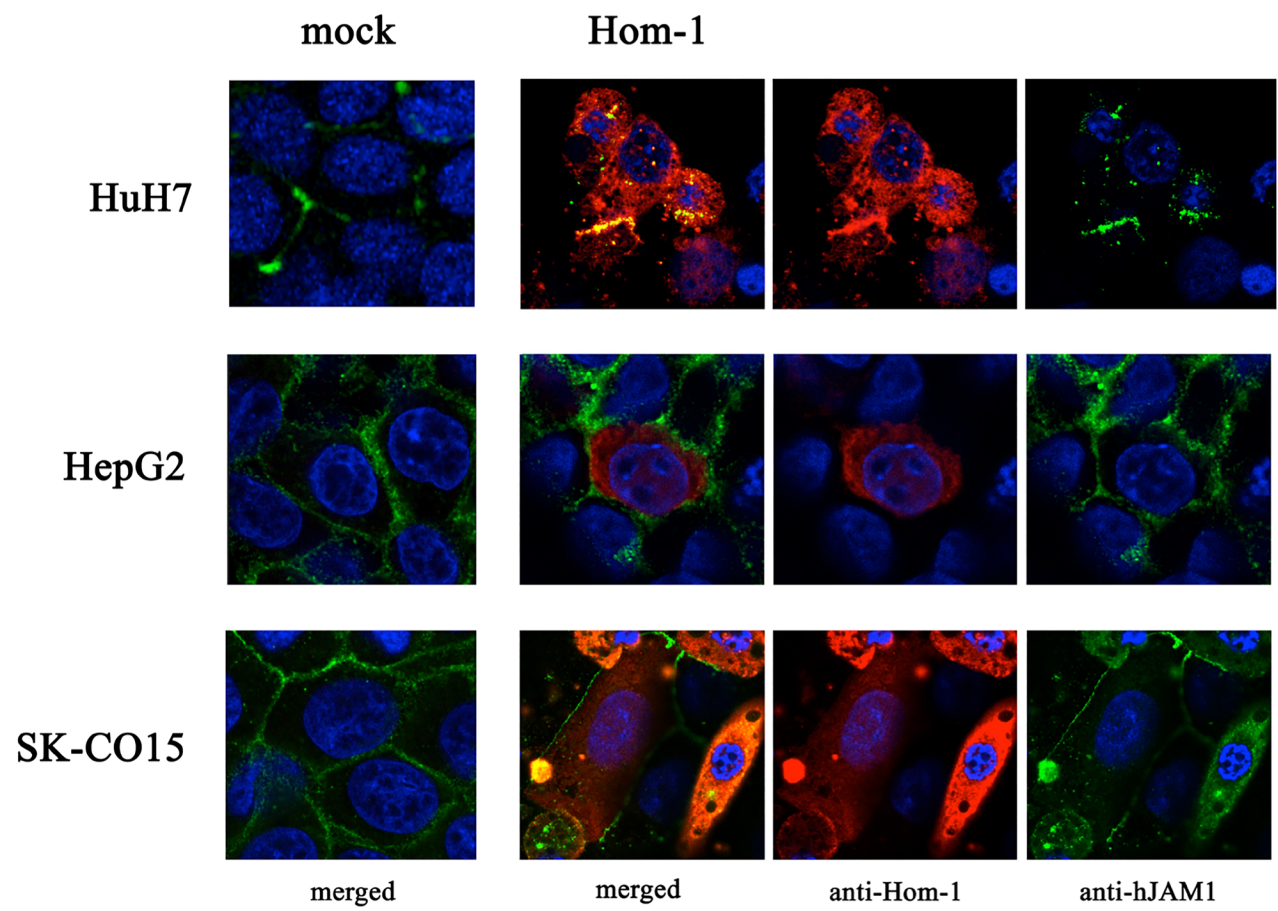

Supplement: FIG S2 [file mbo001173190sf2.pdf]
