# Supplementary material for: Identification of Human Junctional Adhesion Molecule 1 as a Functional Receptor for the Hom-1 Calicivirus on Human Cells
Source: mBio. 2017 Feb 14;8(1):e00031-17. doi: 10.1128/mBio.00031-17 (PMC5312078; doi:10.1128/mBio.00031-17)
Supplement: FIG S3 [file mbo001173190sf3.pdf]

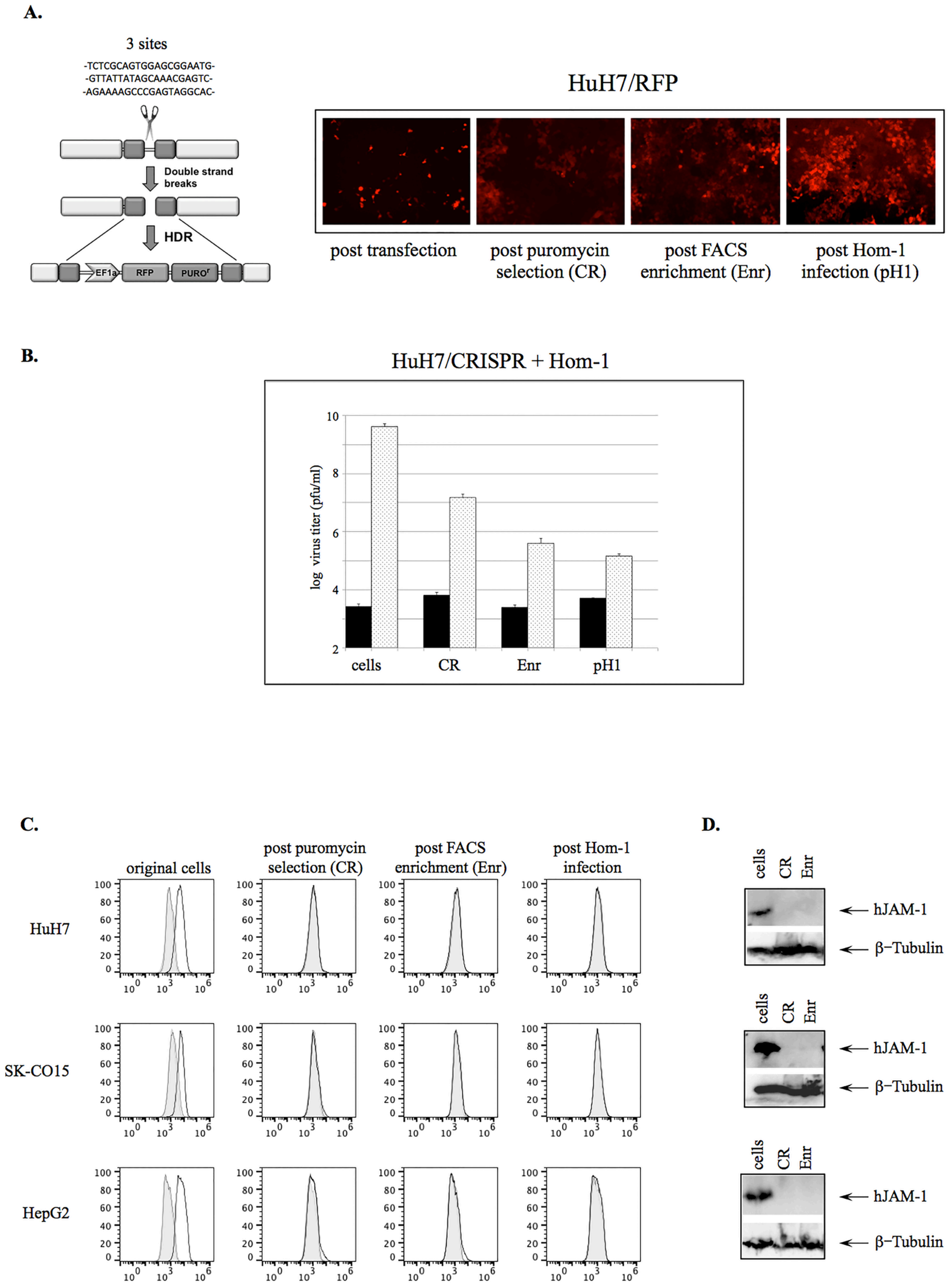

A.
3 sites
-TCTCGCAGTGGAGCGGAATG-
-GTTATTATAGCAAACGAGTC-
-AGAAAAGCCCGAGTAGGCAC-
Double strand breaks
HDR
EF1a
RFP
PUROr
HuH7/RFP
post transfection
post puromycin selection (CR)
post FACS enrichment (Enr)
post Hom-1 infection (pH1)
B.
HuH7/CRISPR + Hom-1
log virus titer (pfu/ml)
10
8
6
4
2
cells
CR
Enr
pH1
C.
original cells
post puromycin selection (CR)
post FACS enrichment (Enr)
post Hom-1 infection
HuH7
SK-CO15
HepG2
D.
cells
CR
Enr
hJAM-1
β-Tubulin
